# Genotoxic and mutagenic effects of polluted surface water in the midwestern region of Brazil using animal and plant bioassays

**DOI:** 10.1590/1678-4685-GMB-2015-0223

**Published:** 2016-10-31

**Authors:** Priscila Leocádia Rosa Dourado, Monyque Palagano da Rocha, Liriana Mara Roveda, Jorge Luiz Raposo, Liliam Sílvia Cândido, Claudia Andréa Lima Cardoso, Maria Aparecida Marin Morales, Kelly Mari Pires de Oliveira, Alexeia Barufatti Grisolia

**Affiliations:** 1 Faculdade de Ciências Exatas e Tecnológicas, Universidade Federal da Grande Dourados, Dourados, MS, Brazil; 2 Faculdade de Ciências Biológicas e Ambientais, Universidade Federal da Grande Dourados, Dourados, MS, Brazil; 3 Universidade Estadual do Mato Grosso do Sul, Dourados, MS, Brazil; 4 Instituto de Biociências, Universidade Estadual Paulista "Júlio de Mesquita Filho", Rio Claro, SP, Brazil

**Keywords:** Allium cepa, Astyanax altiparanae, mutagenicity, cytotoxicity, genotoxicity

## Abstract

This study aimed to evaluate DNA damage in animal and plant cells exposed to water from the Água Boa stream (Dourados, Mato Grosso do Sul, Brazil) by using bioassays, and to identify the chemical compounds in the water to determine the water quality in the area. Through the cytotoxicity bioassay with *Allium cepa*, using micronucleus test, and comet assay, using *Astyanax altiparanae* fish, the results indicated that biological samples were genetically altered. Micronuclei were observed in erythrocytes of *A. altiparanae* after exposure to water from locations close to industrial waste discharge. The highest DNA damage observed with the comet assay in fish occurred with the exposure to water from locations where the presence of metals (Cu, Pb, Cd, Ni) was high, indicating the possibility of genotoxic effects of these compounds. Thus, these results reinforce the importance of conducting genotoxicity tests for developing management plans to improve water quality, and indicate the need for waste management before domestic and industrial effluents are released into the rivers and streams.

## Introduction

The economic development in the 1950s resulted in the territorial and industrial occupation of land that invaded areas of environmental protection, leading to the contamination of water bodies by domestic and industrial effluents. Furthermore, pesticide and chemical inputs contribute to the contamination of streams and rivers located near agricultural regions (Araújo and Dallos, 2006). Water quality and aquatic biodiversity have remarkably decreased because of the exploitation from various human activities that alter the aquatic environment. Examples of such activities include inappropriate land use, effluent discharge, and exploitation by overfishing (Goulart and Callisto, 2003).

The 357/2005 resolution of the Brazilian National Environmental Council (CONAMA) aims to safeguard the water quality of aquatic ecosystems throughout Brazil. This resolution seeks to establish acceptable waste levels of various compounds that are used in domestic, agricultural, livestock, and industrial processes, based on the influence that these compounds can have on physical, chemical, and biological conditions of water.

Biomarkers are used to identify chemicals released into the environment that might cause genetic and chromosomal changes and have direct health effects, leading to human diseases such as cancer, atherosclerosis, cardiovascular disease, and premature aging (Radic *et al.*, 2010). Bioassays performed using microorganisms, animals, and plant cells, separately or in combination with chemical analyses, have been used to define the toxicity of water from various resources (Zegura *et al.*, 2009).

Among the contaminants of urban-industrial origin, high levels of metals are the main compounds that induce toxic, genotoxic, and mutagenic effects in exposed organisms (Wong, 1988). Furthermore, it has been reported that these compounds can induce chromosomal abnormalities, and micronuclei, as well as DNA damage in aquatic organisms (Matsumoto *et al.*, 2006; Barbosa *et al.*, 2010), increasing the ecotoxicological risk.

According to the National Water Agency (ANA, 2009), several agroindustrial activities occur in the vicinity of the Dourados river basin (Dourados, MS) that generate contaminated waste water released in local water resources. The Água Boa is one of several streams located in this basin that receives effluent from domestic sewage and other municipal, industrial and agricultural waste (i.e. neonicotinoids, carbamates, organophosphates, and pyrethroids).

Among the neonicotinoids pesticides used in the state of Mato Grosso do Sul, thiamethoxam and carbendazim were defined as a new class of insecticides widely used against pests in food production and in other numerous purposes, such as in seed treatment and in pets (Banks *et al.*, 2005; Parazajder, 2012, thesis, University of Zagreb, Zagreb, Croácia). The exacerbated use of these products represents a potential threat to humans, since their residues accumulate in food and contaminate aquatic environments. It is, therefore, necessary to intensify studies and promote an efficient environmental monitoring in the region.

The Água Boa stream was monitored using biotests that might allow the identification of possible genetic damage to living organisms due to the presence of pollutants (urban, industrial, and agricultural) that have been deposited in the stream, often without adequate treatment. Thus, this study aimed to identify the chemical compounds present in the Água Boa stream water and evaluate DNA damage in animal and plant cells exposed to these waters. The findings of this study might contribute to the monitoring of water quality in this region.

## Material and Methods

### Characterization of the biomonitoring site

The Água Boa stream is located within the urban perimeter, and larger part of this stream passes across the southern outskirts of the city along the industrial district. Surface water samples were collected in December 2012 and February, April, July, September and October 2013 from three different sites of the stream.

**Figure 1:**
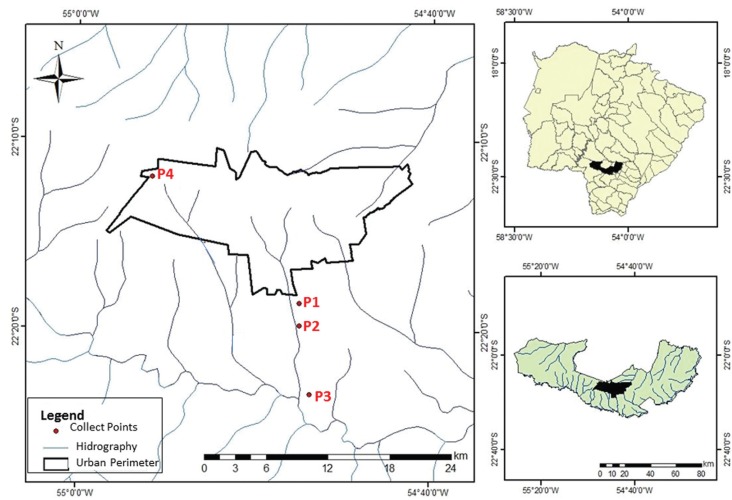
Collection sites for water samples. Collection points from surface water in the Água Boa stream (P1, P2, P3), and groundwater from artesian well of UFGD (P4).

Point 1 (P1: 22.31060 °S, 054.79087 °W) is located about 4.5 km downstream of the urban area; domestic sewage and solid waste are released in this location. Point 2 (P2: 22.32965 °S, 054.79107 °W) is located in the vicinity of the landfill and industrial district, and a near tannery and chicken slaughterhouse are the main factors of water contamination. Point 3 (P3: 22.39558 °S, 054.78407 °W) is located about 0.49 km from the mouth of the Dourados river at the Água Boa stream, which belongs to the Dourados river basin in the Grande Dourados (MS, Brazil) region, and from where water is taken for population supply. This point is impacted by agrochemicals, because of monoculture plantations (corn and soybeans) near its banks. In addition, water samples were collected from groundwater of the Federal University of Grande Dourados (UFGD), namely, Point 4 (P4: 22.19697°S, 054.934458°W), and used to compare the compositions with those of surface water. The sampling site locations (Figure 1) were recorded using a Global Positioning System (GPS).

Water temperature (°C), pH, dissolved oxygen (DO [mg O_2_ L^−1^]), electrical conductivity (Cond [μS/cm^2^]), and total dissolved solids (TDS [mg L^−1^]) were measured using the multiparameter probe HI 9829 (Hanna). Dourados rainfall data were obtained from Embrapa Agropecuária Oeste.

### Water samples

Surface water samples were collected on the same day at around 8:00 am. Water samples used for fish bioassays (*Astyanax altiparanae*) were collected in 20-L polyethylene containers, whereas those for the *Allium cepa* tests were collected in 250-mL polyethylene bottles. These containers were cleaned with tap water before water samples were collected.

For chemical analysis (metals and pesticides), the water samples were collected in 1 L glass bottles. For the determination of metals, the water was acidified with 1 mL acid (65% (v/v), Vetec^®^, Duque de Caxias, RJ, Brazil) and maintained under refrigeration (< 6 °C), whereas samples collected for the determination of organic compounds were frozen at −20 °C.

### Instrumentation, sample preparation and analysis of metals

The protocol used was adapted from the technique described by Raposo Junior *et al.* (2008). An Agilent AA 240FS flame atomic absorption spectrometer (Agilent Technologies, Santa Clara, CA, USA) equipped with hollow cathode lamps was used throughout this work. The operation parameters (such as wavelength, lamp current, slit setting, and air/acetylene flame composition) were adjusted for optimum conditions.

High-purity deionized water obtained using a Millipore Milli-Q Academic^®^ deionizer system (resistivity 18.2 MΩ cm, Millipore, Bedford, MA, USA) and nitric acid (HNO_3_) [65% (v/v), Sigma-Aldrich^®^, St. Louis, MO, USA] were used to prepare all solutions. All solutions and samples were stored in plastic or glass bottles cleaned by soaking in 10% (v/v) HNO_3_ for at least 24 h and thoroughly rinsed in deionized water before use.

For the chemical analysis, 200 mL of water was transferred to an Erlenmeyer flask with reduced to approximately 30 mL, and the final volume was adjusted to 50 mL with 1.0% (v/v) HNO_3_ solution.

Blanks, analytical solutions, and samples were measured in triplicate at the main atomic wavelength for multi-element determination of zinc (Zn), cadmium (Cd), cobalt (Co), chromium (Cr), copper (Cu), iron (Fe), manganese (Mn), nickel (Ni), and lead (Pb).

10 mL HNO_3_, and heated at 90 °C for water preconcentration. The volume was reduced to approximately 30 mL, and the final volume was adjusted up to 50 mL with 1.0% (v/v) HNO_3_ solution.

The following calibration curves were used: 0.0–2.0 mg L^−1^ Zn, 0.2–10.0 mg L^−1^ Cd, 0.2–10.0 mg L^−1^ Co, 0.2–15.0 mg L^−1^ Cr, 0.1–2.0 mg L^−1^ Cu, 0.5–4.0 mg L^−1^ Fe, 0.2–4.0 mg L^−1^Mn, 0.2–10.0 mg L^−1^ Ni, 0.2–15.0 mg L^−1^Pb at a 5.0 mL/min aspiration rate.

### Determination of organic compounds

Two-hundred microliters of the water were subjected to solid phase extraction (SPE) procedure. The process included activation and conditioning of the cartridge with 20 mL methanol and 20 mL ultrapure water, respectively. Subsequently, 200 mL sample was eluted by SPE. The constituents that adhered to the cartridge were eluted with 20 mL methanol, followed by 20 mL ethyl acetate. The methanol and ethyl acetate fractions of each sample were mixed and evaporated. Subsequently, the fractions were diluted in 100 μL methanol, passed through a 0.20 mm membrane filter, and analyzed using high-performance liquid chromatography (HPLC).

#### Liquid chromatography (LC) analysis of standard solutions and samples

For biomonitoring neonicotinoids often used in the fields, the samples and standard carbendazim (CAS No. 10605-21-7) and thiamethoxam (CAS No. 153719-23-4) were analyzed in an LC system (Varian 210, Varian, Sugar Land, TX, USA) with a ternary solvent delivery system equipped with an auto-sampler and a photodiode array detector (PDA) that was monitored at *λ* = 200–800 nm. The LC column was a C-18 (25 cm 4.6 mm; particle size, 5 μm; Luna, Phenomenex, Torrance, CA, USA) that had a small pre-column (2.5 cm × 3 mm) containing the same packing, which was used to protect the analytical column. In each analysis, the flow rate and the injected volume were set as 1.0 mL min^–1^ and 20 μL, respectively. All chromatographic analyses were performed at 25 °C. The samples were eluted using acetonitrile (A): water (B) (65:35) for 5.5 min. The solvent gradient program was as follows: 0 min, 35% B for 5.5 min, 15 min 40% B; 18 min 20% B; and 20 min returning to the initial setting (65% A: 35% B).

#### Linearity

The content of the standard solutions (carbendazim and thiamethoxam) in the samples was estimated using external calibration. Twenty microliters of the dilutions were analyzed using LC, with each determination repeated five times. For the standard solutions, the corresponding chromatogram was obtained, and a graph was constructed from the mean of the chromatogram areas plotted against the standards in 1–100 μg mL^−1^ intervals. A linear least square regression of the peak areas, as a function of the concentrations, was performed to determine the correlation coefficients. The equation parameters (slope and intercept) of the standard curve were used to obtain the sample concentrations.

#### Detection and quantification limits

The detection limits were determined by injecting solutions of thiamethoxam and carbendazim at known concentrations (20 μL each), and then decreasing the concentrations of the samples until a peak with a signal/noise ratio of 3 was detected. The corresponding concentration was considered the minimal detectable concentration. The quantification limit was determined using the same methodology, and was defined as the chromatographic peak with a signal/noise ratio of 10. The organic compound analysis was performed on one sample from P1, P2, and P3 collected in October 2013.

### Biological analysis

#### Plant bioassay

The protocol used was adapted from the described by Matsumoto and Marin-Morales (2005) with modifications. Four plates containing 50 seeds of *Allium cepa* were prepared for tests with water samples (P1, P2, P3, and P4). The seeds were exposed to the water samples for 96 h. The germinated roots were collected and fixed in Carnoy (v/v) 3:1 absolute ethanol/glacial acetic acid for 6 h. Next, the roots were hydrolyzed with 1 mol L^−1^ HCl (37%, Dinâmica, Diadema, Brazil) at 60 °C for 10 min, washed with distilled water and stained with Schiff reagent for 2 h.

For each treatment, five slides were prepared from root meristems and observed under an optical microscope (Nikon 400 objective). From each slide, 1000 cells were counted, totaling 4000 cells per treatment. The calculation of mitotic index (MI) and alteration index (AI) were calculated as follows.

MI = number of dividing cells / number of total cells scored x 100

AI = number of cells with alteration / number of total cells scored x 100

MI and AI averages were subjected to analysis of variance (ANOVA) by using the F test, after data normality was checked using the Shapiro–Wilk test. The parameter means were compared using Tukey's test (significance was set at P ≤ 0.05).

Chromosomal alterations observed in meristematic mitotic cells of *A. cepa* were chromosome breakage, chromosomal adherence, chromosome bridge, chromosome loss, c-metaphase, multipolar anaphases, micronuclei and nuclear bud.

#### Animal Bioassay

The water samples (P1, P2, P3, and P4) were placed in glass tanks (40 × 30 × 20 cm) and aerated at room temperature for 24 h. All fish (*A. altiparanae*) used for the bioassay were provided by the commercial fish farm (Douradense farm). The animal experiments were approved by the ethics committee for animal research of the UFGD, Protocol n°. 005/2013.

Ten fish were placed in each aquarium that had been previously prepared with stream water samples, and maintained for 72 h in the aquaria. Five fish were used for the micronucleus test and the other five were used for the comet assay. Specimens were then collected and anesthetized with 2% (v/v) benzocaine (soluble).

The micronucleus test was performed using blood smear from the tail vein. Two slides from each fish were prepared, fixed in ethanol and stained using Panotic LB. The number of micronuclei (MCN) in the erythrocytes was counted, following the protocol described by Schmid (1975) and Heddle *et al.* (1983), with minor adaptations. In all, 2,000 cells were counted for each fish, and only the cells with intact membranes and cytoplasm were considered for the analysis. The MCN were counted using light microscope (Nikon) at 400× magnification.

The means for MCN were subjected to ANOVA using the F-test, after the data were checked for normality using the Shapiro-Wilk test. Means were compared by Tukey's test (significance was set at p ≤ 0.05).

The comet assay was adapted from the technique described by Ventura *et al.* (2008). Six microliters of blood were collected by puncture and diluted with 2,000 μL of phosphate buffered saline (1:1). For each fish, two slides with 20 μL cell suspension were made and 120 μL 0.5% low melting point agarose 0.5% (v/v) at 37 °C was added. The slides were left in the lysis solution for 1 h at 4 °C. After lysis, the slides were stored in 0.3 mol L^−1^ NaOH buffer and 0.001 mol L^−1^ EDTA (pH > 13) for 20 min. Next, the slides were subjected to electrophoresis at 25 V, 300 mA for 20 min and neutralized with Tris 0.4 mol L^−1^ for 15 min, fixed in ethanol for 10 min and stained with ethidium bromide (0.02 mol L^−1^). In all, 100 nucleoids of each slide (200 nucleoids per treatment/individual) were counted by the same person by using a fluorescence microscope (Labomed; T121100) at 400× magnification.

The nucleoids were classified according to the size of the "tail" and the "head" diameter as follows: class 0, no damage; class 1, low damage level; class 2, intermediate damage level; class 3, high damage level; and class 4, totally damaged. The average frequency of total cells with alterations (TCA) was subjected to the Chi-square test (P ≤ 0.05) to compare the results of the different sites (P1, P2, P3, and P4). The comet assay cells score (CS) was determined by calculating the number of cells with alterations multiplied by the corresponding class (1, 2, 3, and 4).

## Results

The physicochemical analysis of the temperature, pH, oxygen content and total dissolved solids of water samples from all points showed values within the parameters established by CONAMA (2005). However, the values for electrical conductivity showed rates above 100 μS/cm^2^ in all samples.

Rainfall levels in the city of Dourados during the collection period were obtained from the Embrapa Western Agriculture data set (Dourados/MS). The average rainfall for the months (mm) of December 2012 and February, April, July, September, and October 2013 were 3.62, 3.82, 9.59, 4.31, 1.69, 4.23, respectively. The highest and lowest rainfall was recorded in April and September 2013, respectively.

### Chemical analysis

The Cu, Fe, Mn, Cd, Co, Pb and Ni levels in the Água Boa stream water and groundwater samples are shown in Table 1. The water samples collected from P1, P2, and P3 during September and October 2013 contained higher Cu content than the permitted national limit (CONAMA, 2005). However, only P1 and P2 samples collected in April 2013 exceeded the limit of Cu levels. Cd levels exceeded the national standard in P2 (April), P3 (July), and P1 (September), while Pb levels exceeded the national standard in P1 (April and October 2013), P2 (October) and P3 (September and October). Ni levels exceeded the national limits at P1, and P3 from April to October, and P2 from July to October. For all other elements (Fe, Mn and Co), the levels were below the national standard (CONAMA, 2005). Zn and Cr levels were below the detection level at all points. The results from P4 were compared with the 396/2008 CONAMA (2008) groundwater classification regulations, of which Ni levels (from September and October) exceeded the permitted limits.

**Table 1:** Metal levels (mg L^−1^) in the Água Boa stream water samples from April 2013 to October 2013.

Sample	Month	Copper (Cu)	Iron (Fe)	Manganese (Mn)	Cadmium (Cd)	Cobalt (Co)	Lead (Pb)	Nickel (Ni)
P1 (surface water)	April	0.0506*	0.8165	< DL	< DL	0.2461*	0.2565*	0.3740*
	July	#	#	#	#	#	#	#
	September	0.0261*	0.9651	< DL	0.0305*	0.0227	< DL	0.2193*
	October	0.0570*	0.4107	< DL	< DL	0.1957	0.2076*	0.2910*
P2 (surface water)	April	0.0673*	1.218	< DL	0.0468*	0.1123	< DL	< DL
	July	< DL	0.9331	< DL	< DL	0.1004	< DL	0.0740*
	September	0.0274*	1.257	0.0450	< DL	0.1849	< DL	0.2258*
	October	0.0661*	0.6715	0.0190	< DL	0.2533*	0.2377*	0.2959*
P3 (surface water)	April	< DL	0.8110	< DL	< DL	0.1183	< DL	0.0823*
	July	< DL	1.2550	< DL	0.0328*	0.1201	< DL	0.1785*
	September	0.0261*	0.8179	< DL	< DL	0.1843	0.2076*	0.2366*
	October	0.0661*	0.5578	< DL	< DL	0.2227*	0.2189*	0.2502*
P4 (groundwater)	April	0.0223	0.1697	< DL	0.0308	< DL	< DL	< DL
	July	0.0990	0.11	< DL	< DL	0.1147	< DL	< DL
	September	0.0274	< DL	< DL	0.0298	0.2137	< DL	0.1964**
	October	0.0490	0.0930	< DL	0.0202	0.1094	< DL	0.0654**
Permitted levels								
CONAMA 357/2005 (surface water)		0.013	5.0	0.5	0.01	0.2	0.033	0.025
CONAMA 396/2008 (ground water)		2	0.3	0.1	0.005	–	0.01	0.02

Limits of detection (LOD): 0.007 mg L^−1^ Cu, 0.014 mg L^−1^ Fe, 0.005 mg L^−1^ Mn, 0.008 mg L^−1^ Cd, 0.021 mg L^−1^ Co, 0.060 mg L^−1^Pb, 0.018 mg L^−1^Ni. Limits of quantification (LOQ): 0.026 mg L^−1^ Cu, 0.049 mg L^−1^ Fe, 0.017 mg L^−1^ Mn, 0.029 mg L^−1^ Cd, 0.073 mg L^−1^ Co, 0.203 mg L^−1^ Pb, 0.062 mg L^−1^ Ni. DL: Detection Limit

* Values higher than allowed according to CONAMA 357/2005.

** Values higher than allowed according CONAMA 396/2008.

# No sample collected. – Value not found

### Organic compound determination

The organic compound analysis indicated that thiamethoxam was present (Table 2) in the Água Boa stream. As there are no reference values for pesticides in surface waters in the Brazilian legislation (CONAMA 357/2005), we used the limits set by the European community as reference (EC 83/1998).

**Table 2:** Results of organic compounds in the Água Boa stream water (Dourados, MS, Brazil).

Compound (μg L^−1^)	Sampling sites			Permitted levels (μg L^−1^) European Union (83/1998)
	1	2	3	
Thiamethoxam	1.23	1.45	1.58	0.1
Carbendazim	< LOD	< LOD	< LOD	0.1

Limits of detection (LOD): 0.37 μg L^−1^ (thiamethoxam); 0.36 μg L^−1^ (carbendazim). Limits of quantification (LOQ) 1.23 μg L^−1^ (thiamethoxam); 1.20 μg L^−1^ (carbendazim).

### Plant bioassays

The MI values were statistically different between P2 (5.14) and P4 (8.44) in April 2013. P4 was not statistically different from the other three points in any of the other months. At P1, the MI was higher in April (6.72) than in October (1.25). At P2, the highest MI was obtained in February (7.34), and was statistically different from July and October, which had the lowest values. At P3, the highest MI was recorded during February (6.95) and April (6.93), and these values were statistically different from September and October, which had the lowest MI. At P4, the highest MI was recorded in April (8.44), while July, September and October had lower values (Table 3).

**Table 3:** Mean mitotic index (MI) in *Allium cepa* meristematic cells exposed to P1, P2, P3 surface water samples collected from the Água Boa stream (Dourados, MS) and P4 (groundwater) during December 2012 and February, April, July, September, and October 2013.

Collection points	December	February	April	July	September	October
P1	2.79 ± 2.56 ^**a** BC^	4.95 ± 1.53 ^**a** ABC^	6.72 ± 1.50 ^**a** A^	#	5.08 ± 2.39 ^**a** AB^	1.25 ± 1.09 ^**a** C^
P2	4.60 ± 1.23 ^**a** AB^	7.34 ± 2.66 ^**a** A^	5.14 ± 1.46 ^**b** AB^	3.50 ± 1.17 ^**a** B^	4.32 ± 1.21 ^a AB^	1.93 ± 1.30 ^**a** B^
P3	5.31 ± 1.05 ^**a** AB^	6.95 ± 1.36 ^**a** A^	6.93 ± 2.60 ^**a** A^	5.93 ± 1.93 ^**a** AB^	3.22 ± 1.25 ^**a** B^	2.86 ± 1.40 ^**a** B^
P4	5.55 ± 1.90 ^**a** AB^	6.67 ± 2.49 ^**a** AB^	8.44 ± 2.11 ^**a** A^	6.00 ± 2.73 ^**a** B^	2.70 ± 2.7 ^**a** B^	3.33 ± 1.94 ^**a** B^

MI values followed by the same bold lowercase letter in a given column or capital letters in a given row did not differ significantly,

# No sample collected.

P3 had higher AI compared to all other points during April. In all other months, no significant differences were observed among the sampling points. At P1, the highest AI was recorded in February (0.20), and was statistically different from September (0.0). At P3, the highest AI was recorded during April (0.24), which was statistically different from all other months, except September. No significant difference was obtained among months for P2 and P4 (Table 4).

**Table 4:** Alteration Index (AI) in *Allium cepa* meristematic cells exposed to P1, P2, P3 surface water samples collected from the Água Boa stream (Dourados, MS) and P4 (groundwater) during December 2012 and February, April, July, September, and October 2013.

Collection points	December	February	April	July	September	October
P1	0.04 ± 0.06 ^**a** A^	0.20 ± 0.23 ^**a** A^	0.01 ± 0.01 ^**b** A^	#	0 ± 0 ^**b** B^	0.08 ± 0.12 ^**a** A^
P2	0.12 ± 0.11^**a** A^	0.02 ± 0.01 ^**a** A^	0.07 ± 0.13 ^**b** A^	0 ± 0 ^**a** A^	0 ± 0 ^**b** A^	0.07 ± 0.08 ^**a** A^
P3	0.04 ± 0.09 ^**a** B^	0.03 ± 0.03 ^**a** B^	0.24 ± 0.12 ^**a** A^	0 ± 0 ^**a** B^	0.09 ± 0.05 ^**ab** AB^	0.07 ± 0.06 ^**a** B^
P4	0.006 ± 0.01^**a** A^	0.03 ± 0.04 ^**a** A^	0 ± 0.00 ^**b** A^	0 ± 0 ^**a** A^	0.14 ± 0.14 ^**a** A^	0.13 ± 0.13 ^**a** A^

AI values followed by the same bold lowercase letter in a given column or capital letters in a given row did not differ significantly,

# No sample collected.

In surface water samples from P1, P2 and P3, the most frequent chromosomal changes in the meristematic cells of *A. cepa* were in the form of nuclear buds, chromosome bridges, chromosome loss, polyploid cell, C-metaphases, chromosomal adhesions and multipolar anaphase. MCN and chromosome breaks were also found.

### Animal bioassays

#### MCN test with A. altiparanae

In December 2012, fish exposed to water collected from P2 had the highest number of MCN (5.40). Results with statistical similarity were observed in P1 and P3 but not in P4. In February 2013 water from P3 induced the highest average number of MCN (4.20), which was statistically different from those from P4 but similar to P1 and P2 (Table 5).

**Table 5:** Mean number of micronuclei (MCN) in the erythrocytes of *Astyanax altiparanae* exposed to water samples collected from P1, P2, and P3 (surface water) in the Água Boa stream (Dourados, MS) and P4 (groundwater) during December 2012 and February, April, July, September and October 2013.

Average number of micronuclei at collection points						
Collection points	December	February	April	July	September	October
P1	2.80 ± 2.58 ^**a** bB^	3.20 ± 2.48 ^**ab** B^	14.60 ± 11.56 ^**a** A^	#	8.20 ± 4.08 ^**a** AB^	10.60 ± 2.79 ^**a** AB^
P2	5.40 ± 1.94 ^**a** CD^	3.00 ± 2.82 ^**ab** D^	14.80 ± 5.97 ^**a** AB^	15.40 ± 2.70 ^**a** A^	8.20 ± 4.43 ^**a** BCD^	10.40 ± 1.81 ^**a** ABC^
P3	4.20 ± 3.03 ^**ab** B^	4.20 ± 1.78 ^**a** B^	8.80 ± 3.03 ^**a** B^	16.60 ± 2.88 ^**a** A^	9.20 ± 2.16 ^**a** B^	9.20 ± 3.19 ^**a** B^
P4	0.20 ± 0.44 ^**b** B^	0.80 ± 0.83 ^**b** B^	4.60 ± 4.82 ^**a** AB^	3.40 ± 2.19 ^**b** AB^	6.80 ± 3.49 ^**a** A^	3.20 ± 1.48 ^**b** AB^

Average number of MCN followed by the same bold lowercase letter in a given column or capital letters in a given row did not differ significantly by Tukey's test (P ≤ 0.05). Columns indicate statistical differences between collection points, while rows indicate statistical differences between the sampling months.

# Sample not collected

In July and October, a greater number of MCN was observed in fish exposed to the samples from P1, P2 and P3 than from P4. However, during April and September, these points were not statistically different.

The highest number of MCNs was observed in April 2013 (14.60) at P1 and was statistically different from December 2012 and February 2013, which had the smallest number of MCNs. At P2, the highest number of MCNs was observed in July (15.40), which was statistically different from February, September, and December. At P3, the highest number of MCNs was observed in July (16.60), which was statistically different from all the other months (Table 5).

The sources of variation and respective mean squares of MCNs of *A. altiparanae* erythrocytes, and MI and AI observed in the meristematic cells of *A. cepa* are summarized in Table 6. The MCN means were influenced by both collection periods and points, but were not influenced by the interaction between the two. The MI was influenced by all variables (sources of variation and the interaction between them). The rates AI were not influenced by any of the sources of variation.

**Table 6:** Sources of variation, degrees of freedom, and mean square of micronuclei (MCN) number in *Astyanax altiparanae*, in addition to the MI and AI reported in *Allium cepa.*

Sources of Variation	Degrees of freedom			Mean square		
	MCN	MI	AI	MCN	MI	AI
Colection Periods	3	4	4	13.461***	13.541***	0.033^NS^
Colection Points	5	5	5	12.736***	3.352***	0.169^NS^
Periods * Points	14	19	19	0.820^NS^	1.265***	0.119^NS^
CV (%)	24.50	20.15	294.59			
R^2^	0.72	0.78	0.25			

CV coefficient of variation, MI mitotic index, AI alteration index.

*** P ≤ 0.001,

NS not significant (P > 0,5).

#### Comet assay with A. altiparanae

In December 2012, a significantly higher number of total cells with alterations (TCA) was obtained from the water samples (P1, P2, and P3) compared to P4. In the same period, TCA was higher for P3 and was statistically different from P1, which had a higher TCA. In April, P2 had to lowest TCA. In September, TCA values of all three points in the stream were higher than P4. No significant differences in TCA levels were obtained for any of the other months (February, July and October 2013).

In December 2012, significant differences were noted in the number of damaged cells (CS: number of comets number of the corresponding class) at P1, P2, and P3 compared to P4. In addition, the number of damaged cells for P3 was significantly different from P1 and P2. In July, significant differences were obtained for P3 compared to P2. In September, significant differences were obtained for P2 compared to P1 and P3. No significant differences were obtained among any of the points in February, April, and October 2013.

## Discussion

Human activities (agricultural, industrial, and urban) have affected the water quality of the Água Boa stream, causing changes in odor and color along the stream edge at P1 and P2, which are located near the municipal waste and industrial disposal sites. These changes might be related to the release of various chemicals into the freshwater environment (CETESB, 2010).

Electrical conductivity indicates the amount of salts and the concentration of pollutants in the water, and values increase with increased amount of dissolved total solids (CETESB 2010). At all sampling points in the Água Boa stream, the electrical conductivity (above 100 μS/cm^2^) exceeded the permitted limits. The points with the highest electrical conductivity (P2 and P3) were closer to the industrial district, and were located downstream from where industrial effluents were discharged into the water. Thus, the altered conditions of the stream could affected the osmotic balance of animal cells and might explain the changes in *A. altiparanae* when the fish were subjected to the stream water in the aquariums. Although mortality was not recorded in the bioassays, the animals showed difficulty in adaptation (difficulty breathing, increase of gill movement). This fact corroborates the findings of Nunes *et al.* (2011), who suggested that behavioral changes in fish are related to the changes in the physicochemical parameters of water.

The development of economic activities in the last five decades has caused an increase in the concentrations of metals in water resources, impacting the natural environment (Matsumoto and Marin-Morales, 2005). According to Kabata-Pendias and Pendias (1992) and Manzano *et al.* (2014), when the levels of metals are above the national regulation standards, they might become toxic to plants and other organisms. The high metal concentrations in the stream water found in our study might have resulted from the disposal of untreated effluent from the industrial district and agricultural areas.

Small amounts of Cu are essential for the environment under natural conditions. However, excessive quantities could be toxic for fish, microorganisms, and humans (Pereira, 2004). The natural levels of this and other elements in the stream water have increased due to various anthropogenic activities such as the discharge of effluents from sewage treatment, run off from agricultural and industrial processes, and, in some cases, due to high rainfall levels (Rangel *et al.*, 2011). For instance, Cu levels exceeded the national regulations in stream samples during the dry season.

Lead is bioaccumulative and classified as highly toxic for aquatic biota. Even small quantities of this element cause physiological changes in aquatic organisms (Pereira, 2004; Govind and Madhuri, 2014). The Pb content in the Água Boa stream water samples exceeded the national regulations for all samples collected in October 2013 and for P3 samples collected in September (dry season). These results indicate that rainfall affects metal concentrations in the water.

High levels of Ni were also found in the sample and this accumulation affects the behavior of fish because it blocks the gill filaments, causing asphyxiation. In September and October 2013, high Ni levels found in samples collected from the stream could be caused by the low rainfall indexes, mainly in October, and by the disposal of wastes from industries located upstream, leading to the low MI and high CS observed.

High Pb and Ni contents, combined with high electrical conductivity, in the water samples might explain the behavior of *A. altiparanae* observed during the bioassays (Uzoekwe and Oghosanine, 2011).

Cadmium is naturally found in the environment. However, various industrial processes might increase its concentrations, such as electroplating, pigment production, welding, insecticide formulations, incineration of municipal waste and agricultural fertilizers (Farias *et al.*, 2007). In April 2013, Cd levels exceeded the national regulations at P2, and this could be caused by high rainfall rate in April. Thus, fertilizers used for corn culture in this area might have leached Cd to the stream and increased its contamination levels.

The cytotoxity of a compound might be determined by its ability to inhibit or enhance cell proliferation (Fernandes *et al.*, 2007). Metals interfere with MI, induce AI and lead to MCN formation. Thus, metals have mutagenic and cytotoxic potential in plant and animal tissues (Christofoletti *et al.*, 2013). Similarly, we found that high heavy metal (Cu, Cd, Pb, and Ni) levels in September and October 2013 caused a decline in MI in stream samples located near the landfill and industrial district.

Thiamethoxam was detected in the stream samples, indicating that this substance is leached into the stream. This compound is easily dispersed in the soil, and hence has the potential to contaminate aquatic environments (Oliveira *et al.*, 2009). It enters the water because of its use in agricultural crops (maize and sugarcane) in the Dourados region (MS) (ANVISA, 2001). Šojic *et al.* (2012) indicated that thiamethoxam is rapidly metabolized and converted to more toxic by-products. Furthermore, it causes damage to exposed *E. coli* DNA and yields positive results in mutagenicity (Ames Test) and genotoxicity (Comet assay) tests, indicating its possible toxic effect on organisms. Excessive use of such pesticide might contribute to the contamination of water bodies and directly impact the health of aquatic organisms.

Recent studies (Kataeva *et al.*, 2012; Moralejo and Acebal, 2014) have shown high concentrations of heavy metals (Cu, Pb, Cd, Ni) in aquatic environments with pesticide use, leading to changes in cell division processes and chromosome abnormalities. The chemical contaminants in the studied stream water might be responsible for the genetic changes (micronuclei, DNA damage and chromosomal change) observed in the *A. cepa* and *A. altiparanae* bioassays. Thus, the waste from agro-industrial activities might be responsible for genotoxic and cytotoxic properties of the stream water. Similar situations have been documented in the Sava river, Croatia (Radic *et al.*, 2010), Monjolinho river, Brazil (Bianchi *et al.*, 2011) and Pirapó river, Brazil (Peron *et al.*, 2009).

The cytotoxity of a compound might be determined by its ability to inhibit or enhance cell proliferation (Fernandes *et al.*, 2007). The MI analysis in the meristematic cells of *A. cepa* identified cytotoxic, genotoxic, and mutagenic changes in plant tissues treated with water samples from the Água Boa stream. This result might have been caused by the presence the thiamethoxam in the water samples, preventing growth and development of exposed organisms. Furthermore, changes to the MI process led to the emergence of chromosomal changes such as nuclear buds, chromosome bridges, chromosome loss, polyploid cells, C-metaphases, chromosomal adhesions and multipolar anaphases. These changes may or may not be repaired, and are likely to be related to the contamination of the water by heavy metals and pesticides (Majer *et al.*, 2005). This type of damage affects the organization of cytoplasmic microtubules because of the activity of aneugenic substances, which could cause incorrect division and formation of chromosomal changes.

Chromosomal changes might be directly related to the formation of MCN in cells (Bianchi *et al.*, 2011). MCNs are induced by both aneugenic (on the mitotic spindle) and clastogenic (chromosome breakage) actions of chemical agents in the stream environment (Matsumoto *et al.*, 2006).

Recent research has showed an association between high concentrations of metals and erythrocyte damage in tilapia. Thus, the genotoxic effects found in the cells of these animals could be related to the existence of metals in water samples (Duarte *et al.*, 2012). The highest MCN numbers in *A. altiparanae* were documented in our stream samples during April and July 2013. This result indicates the potential of metals (Cu, Cd, Pb, and Ni) to alter the mitotic spindle formation or influence the appearance of chromosomal loss or damage, consequently leading to the emergence of MCNs.

The amount of metals presents in the stream water varied as a function of time (season and period) and collection site. Periods of rain and drought differentially influenced genetic changes in the plant and animal bioassays. MI decreased in the plant bioassays during the dry season (September and October 2013). In contrast, high numbers of MCNs were recorded in the *A. altiparanae* erythrocytes during the rainy period (April and July 2013). These findings corroborate those of a study by Lemos *et al.* (2009).

The genotoxic agents in water samples might cause the loss of DNA integrity, inducing DNA breaks and damage (Nunes *et al.*, 2011). The CS in P3 (Table 7), had the greatest genotoxic potential, particularly when rainfall was low (December 2012 and September/2013), compared with that of P1 and P2, in the high rainfall season. The comet assay was useful to evaluate how chemicals in the water affected *A. altiparanae* erythrocytes, suggesting that the greatest genotoxic effects originated from agricultural and industrial activities. Moreover, the test is sensitive enough to identify DNA damage that may be repaired (Ramsdorf *et al.*, 2009).

**Table 7:** Average total cells with comet alterations (TCA), different comet classes (CL), and cell scores (CS) observed in *Astyanax altiparanae* erythrocytes exposed to P1, P2, and P3 water samples collected from the Água Boa stream (Dourados, MS), in addition to groundwater (P4), during the study months.

Month		Comet classes						CS
		TCA	CL 0	CL 1	CL 2	CL 3	CL 4	
December	P1	64.0 *	44.0	52.4	10.4	1.8	0.0	78.0 *
	P2	54.2 *	60.6	53.0	0.8	0.4	0.0	55.8 *
	P3	92.0 ***	35.4	71.0	19.8	1.2	0.0	114.2 *,**,***
	P4	16.6	85.2	13.0	2.40	1.20	0.0	21.4
February	P1	89.0	27.6	65.0	21.2	2.80	0.0	115.8
	P2	72.8	37.0	58.4	12.0	2.0	0.40	90.0
	P3	90.0	19.2	64.2	23.8	17.8	0.0	117.8
	P4	95.8	17.0	61.2	30.2	4.4	0.0	138.4
April	P1	72.6	33.0	36.0	19.4	14.4	2.8	140.0
	P2	69.4 *	37.6	42.6	15.4	7.8	3.6	112.0
	P3	77.4	32.2	46.2	21.8	7.8	1.6	118.8
	P4	95.8	17.0	61.2	30.2	4.4	0.0	138.4
July	P1	#	#	#	#	#	#	#
	P2	55.6	52.2	37.4	16.6	1.6	0.0	75.0
	P3	77.0	37.4	50.6	23.8	2.6	0.2	106.0 ***
	P4	63.2	50.4	36.2	25.0	1.8	0.2	91.2
September	P1	66.6 *	45.0	41.6	15.0	1.2	0.0	76.4 *
	P2	103.4 *	52.4	37.0	13.4	0.6	0.0	65.6 **,****
	P3	65.4 *	46.4	40.0	17.0	1.6	0.0	78.8 *
	P4	39.8	62.8	32.4	7.2	0.2	0.0	47.4
October	P1	73.4	34.2	42.4	26.6	4.4	0.0	102.0
	P2	67.8	33.0	36.8	24.8	6.2	0.0	105.6
	P3	85.0	24.8	51.2	26.2	7.6	0.0	126.4
	P4	87.2	20.2	56.2	22.2	8.8	0.0	127.0

CL 0: Class 0, no damage; CL 1: Class 1, slightly damaged; CL 2: Class 2, damaged; CL 3 and 4: Class 3; and 4, highly damaged.

* Statistically different compared to P4;

** statistically different compared to P1;

*** statistically different compared to P2;

**** statistically different compared to P3;

# samples not collected

Plant and animal tests showed different responses to contaminants in the stream samples. The greatest damage was observed in *A. cepa* during periods of drought, while MCN levels increased during the periods of rain, as was also reported by Porto *et al.* (2005).

Domestic, industrial and agricultural effluents are potential contaminants of Água Boa stream water. These sources of waste contain different compositions of various substances, such as metals and pesticides, that interact with the cells of organisms, negatively affecting the aquatic biota. Thus, combining the results of chemical and physicochemical analysis for cytotoxicity, genotoxicity, and mutagenicity tests facilitates the accurate monitoring of water quality of many rivers and streams.

This study represents pioneering research in this region that could serve as a basis for the development of future research aimed towards generating information about water quality of rivers and streams throughout Brazil.

In conclusion, cytotoxic, genotoxic and mutagenic damage in *Allium cepa* and *Astyanax altiparanae* might have been found due to the presence of chemical compounds in the water (Cd, Pb, Cu, Ni and thiamethoxam) of the Água Boa stream (Dourados, MS). These compounds, rise an alert to the risk of contamination of the water by other substances, which should be investigated. Thus, considering that the Água Boa stream runs through Dourados city, empties into the Dourados river and is supplied to the population, the biomonitoring of this stream is necessary, to effectively diagnose contamination, as a preventive measure and to mitigate the negative effects of xenobiotics, aiming at the recovery of water quality.
